# Complete Expulsion of Testicular Prosthesis via the Scrotum: A Case-Based Review of the Preventive Surgical Strategies

**DOI:** 10.1155/2015/434951

**Published:** 2015-06-02

**Authors:** Jack Donati-Bourne, A. Deb, Suresh Jay Mathias, Mark Fraser Saxby, Herman Fernando

**Affiliations:** Royal Stoke University Hospital, University Hospitals North Midlands, Newcastle Road, Stoke-on-Trent ST4 6QG, UK

## Abstract

Testicular prostheses are regularly used in urological surgery and are important for postoperative psychological well-being in many patients undergoing orchiectomy. One of the recognised complications of this procedure is graft extrusion, which can result in significant morbidity for patients and require operative reintervention. Whilst most cases of extrusion involve upward graft migration to the external inguinal ring or direct displacement through the scrotal skin, we present an unusual case of complete expulsion of testicular implant three weeks postoperatively through a previously healthy scrotum. During surgical insertion of testicular prostheses, the urological surgeon must carefully consider the different surgical strategies at each step of the operation to prevent future extrusion of the graft. A stepwise review of the preventive surgical strategies to reduce the risk of graft extrusion encompasses the choice of optimal surgical incision, the technique of dissection to create the receiving anatomical pouch, the method of fixation of the implant within the receiving hemiscrotum, and the adoption of good postoperative care measures in line with the principles of sound scrotal surgery.

## 1. Introduction

Testicular prostheses have been employed in urological surgery since the 1940s [1].

They have been shown to be beneficial for men undergoing orchiectomy, particularly in improving perceived body self-image following surgery [2].

Prostheses are not always offered at time of surgery, particularly when orchiectomy is performed for cancer, perhaps due to individual urological surgeons concerns regarding an increased rate of complications and local infection which might result in prosthesis removal and delay in treatment [3].

Recognised complications of insertion of testicular prosthesis include pain, infection, haematoma, scrotal contraction, cosmetic dissatisfaction, and partial extrusion of the graft [4].

Extrusion of the graft may result in significant morbidity for the patient, as well as the need for operative reintervention to remove or substitute the testicular implant.

We present an unusual case of complete expulsion of testicular prosthesis through previously healthy scrotal skin and discuss the preventive surgical techniques to avoid this complication.

To our knowledge there is no report in the literature which comprehensively reviews in a step-by-step fashion the surgical strategies to prevent extrusion of testicular prostheses.

## 2. Case Presentation

PM, a 36-year-old Caucasian male, underwent an uncomplicated day-case elective right inguinal orchiectomy, with primary insertion of prosthesis. He requested the procedure as treatment for longstanding chronic testicular pain, which he had suffered from for ten years following a previous procedure of skeletonisation of the cord.

A right groin incision was made; dissection to the external ring was performed without entry into the inguinal canal. The cord was clamped and transfixed and orchiectomy was performed.

A 34 mm × 39 mm (medium) gel-filled Nagôr Ltd. testicular implant was inserted under strict aseptic conditions.

The implant was not anchored or fixed by any suture material.

3′0 Vicryl was chosen for wound closure and 3′0 Vicryl Rapide for skin.

Histology returned as benign atrophic changes of the right testis.

PM returned to our Surgical Assessment Unit three weeks later reporting right hemiscrotal discomfort postoperatively for two weeks. Subsequently he noticed persistent discharge from an erythematous area of his scrotum where a defect soon developed, and three days later as he kneeled down his testicular prosthesis fell out onto the floor.

He walked in holding the prosthesis in his hand.

Clinical examination revealed a haemodynamically stable and comfortable patient, with a small open scrotal wound which appeared locally infected, and a small cavity where the prosthesis had formerly been.

PM was treated with oral antibiotics and discharged the same day.

He was reviewed in clinic eight weeks later with a well healed wound, declaring the desire to have a new prosthesis reinserted.

## 3. Discussion

The term “extrusion of prosthesis” encompasses both the migration of the implant upwards to the external inguinal ring and the direct displacement through scrotal skin requiring surgical removal. Complete expulsion is unusual and we could not find a case reporting this complication in the literature.

The rate of extrusion of testicular prosthesis is quoted at 3–8% [4]; however, a review performed in the USA of 149 patients undergoing insertion of testicular prosthesis reported extrusion in 2.6% of cases [5].

Risk factors for extrusion include any that increase risk of concomitant infection—such as prosthesis insertion in the presence of epididymo-orchitis, immunosuppression, or diabetes. Previous scrotal surgery and a significant time-interval between initial orchiectomy and implant insertion are also proposed risk factors for extrusion [4].

The sequelae of this complication are discomfort and embarrassment for the patient, as well as the need for further operations and general anaesthesia.

Surgical techniques to reduce the risk of prosthesis extrusion have been explored, although there are no comprehensive reviews of this complication in the literature to our knowledge.

The first surgical consideration, aside from careful patient selection for surgery where possible, is* choice of incision*.

Older reports from urological surgeons proposed the use of a scrotal incision through which the prosthesis could be inserted [6]; however, this practice has fallen out of favour due to higher rates of extrusion of prosthesis [4].

For patients with extensive testicular atrophy, Abbassian proposed the use of a skin incision made in the opposite hemiscrotum not crossing the midline raphe and creating a subcuticular pouch for the prosthesis in the empty hemiscrotum [7]. This procedure is also however, associated with higher risk of implant extrusion.

Lattimer proposed a high scrotal or low inguinal incision to minimise the risk of extrusion of the prosthesis [8], and indeed most surgeons currently would favour a low groin incision to reduce rates of such complications.

Following initial incision, the next surgical consideration is the* creation of the pouch *for the prosthesis.

A common technique is by digital entry into the scrotal sac and the creation of a potential space required for the prosthesis by inflating the balloon of a Foley catheter [9]. This has the advantage of protecting the scrotal neck from excessive stretching during surgery whilst still creating a suitable pouch as the catheter balloon is filled.

Other techniques to create the receiving pouch have been proposed including the use of a vaginal speculum to create the pouch [10] or in children the use of serial Hegar dilators [11]; however, these methods may compromise the required patency of the scrotal neck.

Lawrentschuk and Webb proposed a novel technique for challenging cases or patients undergoing repeat procedures. A low groin incision is used to approach the scrotal neck, identified digitally with fingertip dissection. A pair of Rampley sponge-holding forceps is passed alongside the cord structures into the scrotum, the fulcrum aligned with the scrotal neck so as to not stretch it, and via an opening/closing action the adhesions from previous surgery are divided creating a pouch [12].

The authors claimed that the use of forceps is advantageous over the traditional Foley catheter balloon dilatation technique in that the last 2 cm of dissection—critical to the position of the prosthesis—is reached. Furthermore the rigid forceps can be directed to the desired site whilst the nonrigid Foley catheter may not be able to negotiate past dense surgical adhesions.

Lawrentschuk and Webb reported no events of prosthetic extrusions in their case series of ten patients undergoing this procedure and described how in their opinion this was because their technique prevented overstretching of the scrotal neck which may lead to prostheses migration [12].

There are no studies in the literature which compare the rates of extrusion associated with the different proposed surgical techniques of testicular prostheses pouch creation.

After the anatomical pouch has been created, the surgeon must carefully decide on which* type of implant* to use.

A material for prostheses must be chemically inert, not cause an inflammatory reaction, resist mechanical strain, be amenable to sterilisation, and ideally feel natural for the patient. Currently the vast majority of testicular prostheses are made of silicone-based gel or are saline-filled [9].

There is no particular type of testicular implant which is associated with a higher rate of extrusion. However, previous concerns, with regard to silicone implants increasing the risk of malignancy and connective tissue diseases which temporarily led to a suspension in their use, have not been substantiated [13].

There are no studies in the literature which compare the rates of extrusion associated with the different types of testicular implants.

A key element during the operation that can affect extrusion rate is the* technique of implant fixation.*

The most common technique to secure the implant within the hemiscrotum is to invert the scrotum and secure the prosthesis with a PDS suture placed through the suture loop present on most implants. Particular attention must be paid to not take the suture bite too deep to avoid skin penetration which may promote infection and subsequent implant extrusion [9].

This technique was disputed by Yossepowitch et al. in their report of 86 patients receiving testicular prosthesis between 1995 and 2009. The authors described using in their earlier years a nonabsorbable suture to secure the prosthesis; however, following a large number of patient complaints regarding the implant's high upward migration, they chose to abandon the practice of transfixation thus allowing the prosthesis to move naturally within the scrotum [14].

There are several surgeons, however, who do not routinely fix the implant to the scrotum in cases where this is technically difficult but rather rely on the postoperative adhesions to hold the prosthesis in place.

In Zilberman et al.'s case series of nineteen paediatric testicular implant recipients, three patients did not have theirs sutured to the lower scrotum, and no postoperative complications were reported in any of their patients [15].

Ferro et al. described a technique used in children where the prosthesis is slid into the scrotum and kept in position by closing the scrotal entrance with a single suture through a small skin incision at the scrotal root [16].

*Postoperative care measures* are important in the final aspect of preventing testicular implant extrusion and rely on the basic principles of scrotal surgery, including the use of scrotal support wear, prophylactic antibiotics in high-risk cases, and advice to avoid lifting heavy weights.

Despite undertaking all of the above precautions, the attending surgeon must counsel all patients undergoing insertion of testicular prostheses of the risk of extrusion of the graft.
